# Mortality of Lowell in 1845

**Published:** 1846-04

**Authors:** 


*Mortality of Lowell in* 1845.—Dr. Wells, the City Physician of Lowell, states that "the number of deaths reported during the last year was 363, differing but one from either of the two previous years. This must be regarded quite satisfactory, as indicating, when we consider the increase of population, a diminished mortality.

"In the course of the last year the small pox has made its appearance five times, but without proving fatal in any case, and in but one instance has it extended beyond a single case. In other places similarly situated, this loathsome disease has prevailed extensively and fatally; and our exemption, under the circumstances, can only be accounted for by the efficient measures that have been taken, to secure a general vaccination of the inhabitants.

"Of the efficacy of vaccination as a means of guarding against small pox, there can be but one opinion; and there is good reason to believe, that equal exemption from varioloid might be secured, if vaccination were repeated as often as necessary.

"It is to be regretted that a prejudice, unfounded should prevent some persons from availing themselves of its advantages. That vaccination may, in constitutions strongly predisposed, prove the *exciting* cause of certain diseases, is not impossible; but that it operates in accordance with common prejudice, by transferring what are termed *humors*, from one to another, my experience in about 2000 cases leads me to disbelieve."—*Ibid.*

